# Edible Brown Seaweed in Gluten-Free Pasta: Technological and Nutritional Evaluation

**DOI:** 10.3390/foods8120622

**Published:** 2019-11-27

**Authors:** Patrícia Fradinho, Anabela Raymundo, Isabel Sousa, Herminia Domínguez, María Dolores Torres

**Affiliations:** 1 LEAF–Linking Landscape, Environment, Agriculture and Food, Instituto Superior de Agronomia, Universidade de Lisboa; Tapada da Ajuda, 1349-017 Lisbon, Portugal; anabraymundo@isa.ulisboa.pt (A.R.); isabelsousa@isa.ulisboa.pt (I.S.); 2 Department of Chemical Engineering, Universidade de Vigo (Campus Ourense), Science Faculty, As Lagoas, 32004 Ourense, Spain; herminia@uvigo.es (H.D.); matorres@uvigo.es (M.D.T.)

**Keywords:** kombu, *Psyllium*, *Laminaria ochroleuca*, by-products, celiac, cooking quality, antioxidants, fibre, minerals

## Abstract

Seaweed is a novel source of important nutritional compounds with interesting biological activities that could be processed into added-value products, namely gluten-free foods. In this study, two previously developed products obtained from *Laminaria ochroleuca* processing (liquid extract and a purée-like mixture) were incorporated in gluten-free (GF) pasta in order to develop functional products especially designed for the celiac population. The raw and cooked pastas were characterized in terms of their cooking quality parameters, nutritional composition, texture and rheological properties, and antioxidant activity. It was found that the developed GF pastas had similar mechanical and texture characteristics to the control. Both supplemented GF pastas presented a significantly (*p* < 0.05) higher fibre and mineral content than the control pasta.

## 1. Introduction

Innovative food products with health-promoting components have gained growing interest within the research community, food industry, and consumers [1,2,3,4]. The gluten-free (GF) foods available in the market are nutritionally unbalanced, promoting high intake of energy, sugars, lipids, and a low intake of fibre, vitamins (B group and D), and minerals (Ca, Fe, Mg, and Zn) [5,6]. Besides nutritional deficiencies, celiac consumers consider GF foods less appealing in terms of texture and flavour than similar products with gluten [7].

Seaweed is a natural source of interesting compounds for human nutrition, such as fatty acids, proteins, minerals [8], vitamins (A, C, D, B group, E, K, PP) [9], and pigments such as carotenoids and polyphenols, with proven antioxidant, hypoglycaemic, antitumoral, and antimicrobial activities [10,11,12].

Although their consumption is not massified, their popularity has increased, especially amongst vegetarians and consumers looking for new food sources and tastes [13]. A challenge for the coming years is to introduce seaweed or their high-valuable extracts as additives in food matrices. Due to its high content in biologically active compounds, seaweed, especially Phaeophyta (brown algae), has great potential to be used in food applications [14,15]. However, despite the interest of the research community and the flourishing availability of Asian food, the current European retail market of seaweed food products is limited to canned food, salad dressings, and pâtés, among others [16,17]. 

*Laminaria ochroleuca* is a perennial warm-water temperature brown seaweed, distributed from the south of England to Morocco [18]. It has interesting biochemical composition [19] and could enhance the nutritional profile of food products targeted for populations with specific nutritional requirements such as the ones of the celiac population and consumers with some type of wheat allergy.

Several studies of seaweed incorporation and/or their extracts into several food matrices (meat, pasta, cheese) have been performed [20,21,22] to enhance nutritional and texture features, antioxidant capacity, or shelf life stability of the product. Regardless of the potential already described, to our knowledge, there are no available studies addressing the supplementation of GF foods with seaweed.

Processing conditions modify the nutritional and mechanical profile of foods, either by contributing to the acquisition of desirable features (e.g., crunchiness), or to the loss of antinutrient phytochemicals, as well as some losses of vitamins and minerals [23]. Moreover, processing methods improve protein and carbohydrate digestibility of foods [24,25,26]. Therefore, the assessment of the actual nutritional and phytochemical composition of foods is a mandatory issue for the design of functional foods for populations with specific requirements.

This study aims to add value to *L. ochroleuca* brown algae, through its incorporation as a high-valuable additive in functional GF pasta. The raw and cooked GF pastas were extensively characterized in terms of physical properties and biochemical composition, in order to assess the actual nutrient intake.

## 2. Materials and Methods

### 2.1. Raw Materials

Dehydrated *Laminaria ochroleuca* (Algas Atlánticas Algamar, S.L., lot B39137547, Pontevedra, Spain) was milled and sieved to obtain powders with <0.25 mm and 0.25–2 mm diameter particle sizes. Rice flour (Ceifeira, Dacsa Atlantic, lot 3411/18, Lisboa, Portugal) and *Psyllium* husk of Indian origin (Solgar, lot 107028-01, Leonia, NJ, USA) were purchased in a local market. *Psyllium* was milled and sieved to 160–315 μm (Pulverisette 14 Premium, Fritsch, Idar-Oberstein, Germany).

### 2.2. Fresh Pasta Preparation and Sampling

**Figure 1:**
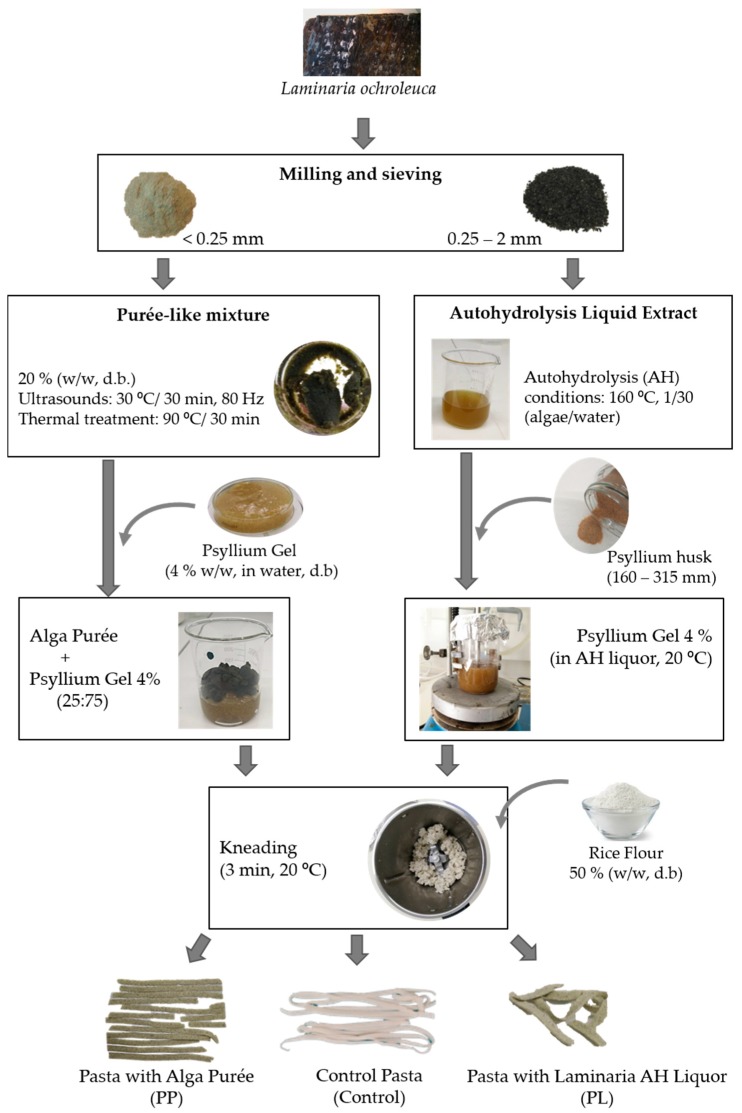
Schematic procedure of pasta preparation. dry basis: (d.b.)

Focusing the full valorisation of *L. ochroleuca*, two samples were prepared for pasta incorporation: (1) autohydrolysis (AH) liquid extract obtained according to the procedure described in Fradinho et al. [19]; (2) purée-like mixture (20% (*w/w*) alga, ultrasound 30 min, 90 °C/30 min prepared following the procedure already described by Fradinho et al. [19] (Figure 1).

Then, both the AH liquid extract and the purée samples were processed with *Psyllium* husk or *Psyllium* gel (4% *w/w*, dry basis (d.b.)) [27] to obtain *Laminaria–Psyllium* gels. The selection of *Laminaria–Psyllium* 25:75 ratio was on the basis of absence of syneresis and good mechanical properties assessed in a previous study by the authors [28].

Three batches of 200 g of each pasta formulation were prepared by mixing each *Laminaria–Psyllium* gel with rice flour (thermoprocessor: Bimby TM31, Vorwerk, Wuppertal, Germany), at a 50:50 ratio, at room temperature for 3 min.

The dough was sheeted and laminated as tagliatelle (width = 6.10 mm, thickness = 2.12 mm, length = 70 cm) using a benchtop pasta machine (Atlas 150 Wellness, Marcato, Campodarsego, Italy). Then, the pasta dough was covered with aluminium foil and placed in an air oven at 25 °C for 15 min to ensure structure recovery. A control sample was also prepared using only rice flour and *Psyllium* gel 4 % (*w/w*).

Cooking quality of pasta (optimal cooking time (OCT), water absorption, swelling power, and cooking loss) was assessed on raw samples following the procedure described in AACC 66–50 [29] and the methods described in Fradinho et al. [30]. Each determination was performed at least three times.

The pasta samples (uncooked and cooked) destined for physicochemical and bioactive analysis were lyophilized at −55 °C for 72 h (Scanvac Coolsafe 55-4, Labogene, Allerød, Denmark), crushed into powder (<0.5 mm), and stored at room temperature until biochemical analysis. Physical analyses (colour, texture, and rheology) were performed on fresh samples immediately after pasta resting.

### 2.3. Colour Evaluation of Pasta

The colour measurements of raw and cooked pasta samples were performed instrumentally using a CR-400 colorimeter (Minolta, Tokyo, Japan) with standard illuminant D65 and a visual angle of 2°. For calibration purposes, a white standard was used (L* = 97.21; a* = 0.14; b* = 1.99). The colour parameters (L*, a*, and b*) were accessed by CIELAB system and the total colour difference between raw and cooked samples was calculated according to Equation (1).

ΔE* = (ΔL* + Δa* + Δb*)^1/2^. (1)

The measurements were conducted at 20 ± 1 °C and replicated at least eight times.

### 2.4. Physicochemical Analysis of Pasta

Moisture content was determined gravimetrically on raw, cooked, and lyophilised samples using an automatic moisture analyser PMB 202 (aeADAM, Milton Keynes, UK) at 130 °C, until constant weight.

Ash content was determined gravimetrically by incineration at 550 °C in a muffle furnace (Snol LHM01, Utena, Lithuania). Protein analysis was performed by the Kjeldhal method according to the ISO 20483 [31] for cereal and pulses. The determined total nitrogen content was multiplied by a conversion factor of 5.95 [32] to obtain the pasta crude protein content.

The total lipids content was determined according to the procedure used for cereals and derived products in the Portuguese standard method NP4168 [33], as described in Batista et al. [34].

Soluble, insoluble, and total dietary fibre contents of *Laminaria ochroleuca* and all pasta samples (raw and cooked) were evaluated according to AOAC 991.43 [35] with the specific modifications for *Psyllium* fibre suggested by Lee et al. [36].

Minerals presence and content were carried out by inductively coupled plasma (ICP) spectrometry (iCAP Spectrometer equipped with ASX-520 AutoSampler(Thermo Scientific, Waltham, MA, USA)). Briefly, 0.25 g of lyophilized sample was weighed, transferred to digestion vessels, and 9 mL of HCl and 3 mL of HNO_3_ were added. The digestion (SCP Science, DigiPREP MS, Baie d’Urfe, QC, Canada) took place at 30 min/40 °C, 30 min/80 °C, and 90 min/105 °C. After cooling, distilled water was added up to 50 mL, and the solution was left to decant. Finally, the clear supernatant was used in ICP analysis. Eleven elements (Na, K, Ca, Mg, P, S, Fe, Cu, Zn, Mn, I) were determined in each sample, and were selected on the basis of their significance in the study of GF food. All chemical composition analyses were repeated at least in triplicate.

### 2.5. Phytochemicals and Antioxidant Activity Measurements

Raw and cooked pasta samples were subjected to extraction (in duplicates) according to Sant’Anna et al. [37]. Briefly, 1 g of lyophilized sample was mixed with 50 mL of a mixture of ethanol/water (50:50), incubated at 60 °C for 1 h under magnetic stirring, and then filtrated with Whatman filter paper n.1. The liquid extracts were recovered and used for total antioxidant activity (AA) and total phenolic content (TPC) measurements.

All the following spectrophotometric methods were performed in triplicate in a Unicam UV4 spectrophotometer (Thermo Scientific, Waltham, MA, USA).

ABTS radical (2,2-azinobis(3-ethyl-benzothiazoline-6-sulfonate)) scavenging was performed according to Re et al. [38]. A mixture of ABTS ^+^ working solution (3 mL) and the liquid extracts (30 μL) described above were incubated at 30 °C for 6 min (A_sample_). The absorbance (A) was measured at 734 nm, and the percentual absorbance reduction regarding the initial value (% RSA) was determined according to Equation (2).

RSA (%) = (A_Blank_ – (A_sample_ − A_control_))/A_Blank_ × 100. (2)

Two blank assays, one without sample (A_Blank_: phosphate buffered saline buffer) and another without reagents (A_control_) were performed simultaneously.

A calibration curve with Trolox aqueous solutions (0.1–1 mM) was made, and the results were expressed as millimole TEAC (Trolox equivalent antioxidant capacity) per gram of sample (dry basis).

The antioxidant activity against DPPH (2,2-diphenyl-1-picrylhydrazyl) radical was also measured. In this case, 50 μL of each liquid extract was mixed with 2 mL of the DPPH radical working solution (6 × 10^−5^ M), and was incubated, in the dark, at room temperature for 16 min. The absorbance of the samples, control, and blank was measured at 515 nm. Results were expressed as described for the ABTS procedure.

The total phenolic content (TPC) was determined by the Folin–Ciocalteu method. Briefly, 250 μL of sample, 1875 μL of deionised water, 125 μL Folin–Ciocalteu (1 M), and 250 μL of Na_2_CO_3_ (10%, *w/v*) were mixed and incubated for 1 h in the dark at room temperature. Absorbance was measured at 765 nm against water. Gallic acid (20–120 mg/L) was used as a standard for quantification, and the results were expressed as milligram GAE (gallic acid equivalent) per gram of sample (dry basis).

### 2.6. Mechanical Characterization of the Pasta

Texture measurements were performed on raw and cooked pasta samples, in a TA-XT plus texturometer (Stable Microsystems, Godalming, United Kingdom) with a 5 kg load cell in a 20 °C controlled temperature room.

Texture profile analysis was performed on all raw pasta samples according to the method already described in Fradinho et al. [30]. Regarding cooked pasta, compression and tension tests were performed. Before each test, pasta samples were cooked in boiling water during optimal cooking time, rinsed with distilled water, and drained. Three types of texture measurements were performed:

- Cutting: the firmness of cooked pasta samples was measured following AACC method 66–50.01 [29]. Pasta firmness (N) was determined by measuring the cutting force required to cut three cooked tagliatelle strips using a blade set with guillotine (HDP/BSG) that cut 2.5 mm into the sample at 0.17 mm/s. From this test, adhesiveness (N·s), which is the resistance of the material when the probe is recessing, was also measured.
- Stickiness: pasta stickiness was defined as the maximum peak force required to separate the probe from the sample surface (peak height), and the area under the peak represented the work of adhesion. Three tagliatelle strips were centrally aligned under a circular plexiglass probe (44 mm diameter) on a raised platform and were retained within a circular slot (48 mm diameter) made in a base plate. The samples were compressed for 2 s with an applied force of 9.807 N at 0.5 mm/s. The precision of the stickiness measurement decreased as elapsed time increased. Therefore, the time for stickiness measurements was set at 15 min after draining.
- Extensibility: cooked pasta extensibility characteristics were determined using a Kieffer Dough and Gluten Extensibility Rig (A/KIE). Sample loading and test were conducted as follows: a tagliatelle strip was placed across the grooved region on the sample plate. The hook probe was positioned under the strip and then raised upward at 2.0 mm/s, stretching the strip until rupture. From this tension test, two parameters were obtained: the maximum resistance to extension (Rmax, N) and the extensibility until rupture (ERmax, mm).

Each test was replicated at least seven times in each pasta formulation.

Dynamic oscillatory rheology measurements were used to monitor the viscoelastic characteristics of raw and cooked pasta samples. After pasta preparation, the dough was divided into two fractions: one portion was immediately tested, whereas another portion was cut into circular disks (30 mm diameter, 2 mm height) and cooked at OCT before testing. Small amplitude oscillatory shear testing was conducted at least in duplicate in a controlled stress rheometer (MARS III, Haake, Karlsruhe, Germany) using serrated parallel plate geometry (PP 20:20 mm diameter) to avoid the slip effect and 2 mm gap (previously optimized). Surface geometry was covered with paraffin oil to prevent moisture loss. Samples were rested before rheological testing to allow temperature equilibration (5 min, 20 °C—previously optimized). Then, stress sweep tests were run at 1 Hz from 0.1 to 100 Pa to assess the linear viscoelastic region (LVR). Finally, the mechanical spectra were performed through frequency sweep tests from 0.1 to 10 Hz (20 °C, 10 Pa) within the LVR previously defined for each sample.

### 2.7. Statistical Analysis

The experimental data was statistically analysed using RStudio (Version 1.1.463 2009–2018 RStudio, Inc., Boston, USA). Analysis of variance (one-way ANOVA) was used to assess significant differences between samples at a significance level of 95% (*p <* 0.05). Multiple comparisons were performed by Tukey HSD (honestly significant difference) est. All results are presented as average ± standard deviation (s.d.).

## 3. Results and Discussion

### 3.1. Pasta Cooking Quality

**Figure 2:**
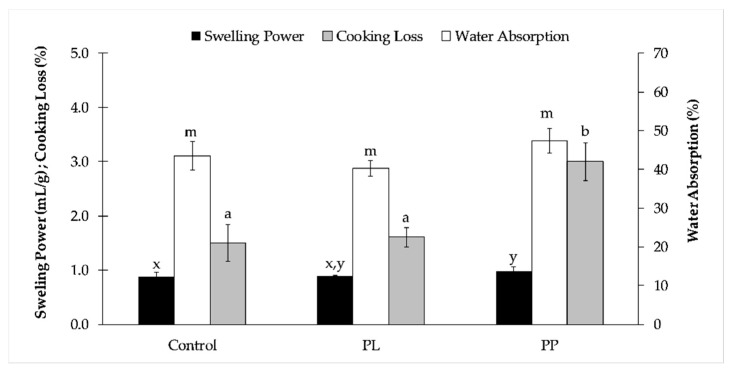
Cooking quality parameters of pasta prepared with *Laminaria ochroleuca* liquid extract (PL), alga purée (PP), and control (without alga). Data are presented as mean ± standard deviation (s.d.). Different letters in the same parameter correspond to significant differences (*p* < 0.05, one-way ANOVA, *post-hoc* Tukey test).

All pasta samples presented the same optimal cooking time—2 min. The results of the pasta quality performance upon cooking are shown in Figure 2.

Control and *L. ochroleuca* liquid extract (PL) pastas present similar cooking behaviour. Pasta prepared with *Laminaria* purée (PP) showed significantly (p < 0.05) higher swelling power than the control, probably due to the alga capacity of retaining the cooking water.

Due to the lack of the gluten network, gluten-free (GF) pastas rely mostly on starch for creating the matrix and entrapping the water. Thus, GF pastas generally show higher cooking loss (CL) than wheat pastas [39]. However, all GF pastas developed here showed lower CL than durum wheat spaghetti commercial samples reported by Bonomi et al. [40], proving their quality upon cooking. The higher CL of PP could be related to the higher fibre content of this pasta (Table 1), and also with its lower *Psyllium* content—(1.5% (*w/w*) against 2% (*w/w*) of the other formulations), which has a high water absorption capacity at about 1 g *Psyllium*/15 g water [41].

### 3.2. Colour Stability upon Cooking

**Figure 3:**
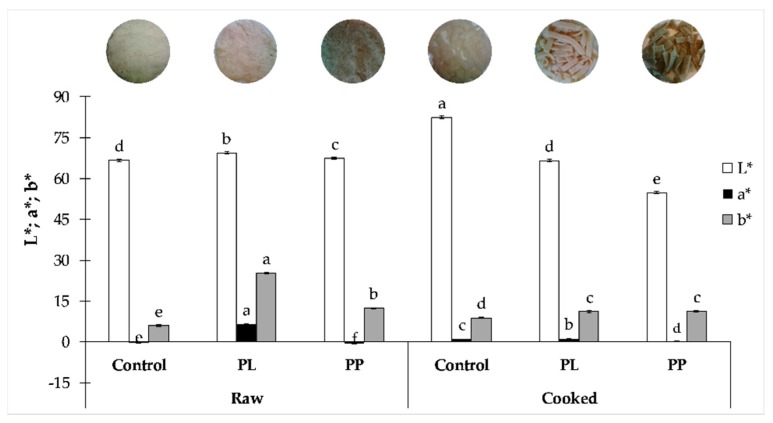
Colour parameters (L*, a*, b*) of raw and cooked pastas. Different letters in the same parameter (e.g., L* raw and cooked) correspond to significant differences (*p* < 0.05, one-way ANOVA, *post-hoc* Tukey test).

Colour stability is an important attribute when working with colourful food products. In Figure 3, the results of colour parameters obtained in raw and cooked pastas are shown.

As expected, the addition of *L. ochroleuca* changed the colour of the GF pasta, ranging from the whitish colour of control pasta, to green of PP, reflecting the use of the whole alga in the formulation. The most noticeable feature was the colour difference between raw and cooked pastas (12.8 ≤ ΔE* ≤ 16.1). These results mean that the thermal processing applied to the GF pastas was responsible for a significant (*p* < 0.05) modification of the sample’s colour, meaning a pigment loss (leaching). However, a lower ΔE* was observed in PP pasta (with alga purée), which could indicate the resistance of the product to the thermal procedure applied, as already observed in pasta with microalgae incorporation [42].

**Figure 4:**
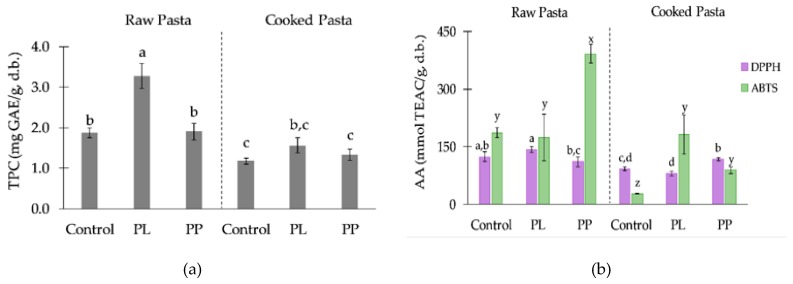
Total phenolic content (**a**) and antioxidant activity (**b**) of raw and cooked pastas: with *L. ochroleuca* liquid extract (PL), alga purée (PP), and control (without alga). Different letters in the same parameter correspond to significant differences (*p* < 0.05, one-way ANOVA, *post-hoc* Tukey test). DPPH: 2,2-diphenyl-1-picrylhydrazyl, ABTS: 2,2-azinobis(3-ethyl-benzothiazoline-6-sulfonate), TPC: total phenolic content, GAE: gallic acid equivalent, AA: antioxidant activity, TEAC: Trolox equivalent antioxidant capacity.

Regarding a* and b* colour parameters, both PL and PP showed a significant (*p* < 0.05) decrease in redness and yellowness, probably associated with pigment loss during cooking (brownish tonality of the cooking water), especially fucoxanthin and phlorotannins, major pigments of brown algae [10,43]. These results are supported by the decrease of phenolic compounds and antioxidant activity in cooked pastas (Figure 4) and the report by Prabhasankar et al. [21] for wheat pasta supplemented with *Undaria pinnatifida*.

### 3.3. Physicochemical Analysis of Pasta

In Table 1, the chemical composition of raw and cooked pasta samples is presented. 

Celiac disease is an auto-immune disorder characterized by malabsorption of nutrients by the small intestine, leading to multiple nutritional deficiencies [44]. A gluten-free diet is a lifelong therapy for the celiac population and gluten-sensitive individuals. However, GF foods are very often nutritionally unbalanced, providing excess calories, and being high in lipids and low in fibre content [5,45,46]. The pastas developed in the present study showed very low lipid content (as *Laminaria ochroleuca* has only about 7% (*w/w*) lipids, 55% of which were unsaturated fatty acids [47] and just 0.8% (*w/w*) of lipids for the rice flour [30]. *Laminaria* incorporation (either liquor or purée) contributed significantly (*p* < 0.05) to the protein increase in pasta. The energy value was also lower—*Laminaria* pastas showed values between 202–207 kcal/100 g (fresh weight), much lower than the energy value (369 kcal/100 g, fresh weight) reported for semolina pasta with *Undaria pinnatifida* [21], and 310–361 kcal/100 g for commercial GF dry pasta [45,48].

*Laminaria* addition significantly increased (*p* < 0.05) the insoluble fraction of dietary fibre. Seaweed’s high fibre content is responsible for its capacity to absorb and retain water [49], and it can be used as a source of dietary fibre in bread [50] and noodles [51], also improving the mineral content. In general, seaweed can be used as a texturing and bulking agent in low-calorie foods (e.g., meat emulsions [20]).

One of the main features of *Laminaria ochroleuca* is its mineral content, mainly K, Mg, I, and Ca [19]. Although in some cases 40% of the minerals were lost during the cooking of pasta, the amount that remained after thermal processing was still high, and some of the mineral elements were not lost during cooking (Ca, S, Zn and Mn). This alga also showed 413 mg/100 g (d.b.) and 676 mg/100 g of iodine in the liquid extract obtained by autohydrolysis [19], and even if some iodine was lost during cooking, iodine presence remained strong.

Regulation (EC) 1169/2011 [52] on the provision of food information to consumers sets that 15% of the recommended daily allowance (RDA) of each mineral should be taken into consideration when deciding what constitutes a significant amount (Table 2). Also, nutrition claims are assessed on the basis of the intake of the specific nutrient in the ready-to-eat food product. All cooked pastas showed less than 0.5 g lipids/100 g, and PP pasta had more than 3.0 g/100g of fibre content. Therefore, according to the regulation (EC) No. 1924/2006 [53] amended by regulation (EU) no. 1047/2012 [54] about the nutrition claims, all pastas can bear a claim as being “fat-free”, and PP pasta could also have a claim as a “source of fibre” for products containing at least 3 g dietary fibre/100 g product, which is an added value in terms of commercialization of these *Laminaria* pastas.

As the impact of the continued ingestion of nutritionally unbalanced GF foods on the health of consumers is countless, contributing to the development of associated diseases, such as chronic constipation, type 1 diabetes, thyroid malfunction, anaemia, and overweight [55,56,57,58], these pastas are a breakthrough in terms of specially designed foods for this target population.

Commercial GF cereal foods, made of refined flours or starches, are also of lower nutritional value compared to their wheat counterparts [59]. Considering the average concentrations of some mineral elements found in the present study and a daily amount of 100 g of pasta, one can conclude that it provides more for the recommended daily allowance (RDA) of minerals than GF pasta without *Laminaria*.

As mentioned earlier, the most deficient minerals in a GF diet are Ca, Fe, Mg, and Zn. In a review, Rondanelli and co-workers [60] gathered suggestions and guidelines for supplementation with several minerals and Theethira et al. [55] reported a reduction in calcium intake by about 76%–88% of patients adhering to a GF diet. Although *Laminaria ochroleuca* is a good source of Ca, the PP pasta only contributed to 1.9% of RDA for calcium, whereas Ca supplementation in the range of 1200–1500 mg/day is recommended. The importance of calcium and vitamin D should also be taken into account considering the prevalence of metabolic bone diseases in the celiac population, and some authors [9] reported the presence of vitamin D in *Laminaria ochroleuca*, which is determinant for Ca absorption.

Iron is an essential trace element, being mostly bound to hemoglobin in circulating erythrocytes, and the amount of iron in the body depends mainly on its absorption [61]. Iron deficiency is very common in celiac disease patients, leading to anaemia, which affects up to 80% of newly diagnosed celiac patients, and pasta with *Laminaria* purée caused a significant (*p* < 0.05) increase in iron content (Table 1), equivalent to 10.3% RDA.

Zinc deficiency affects about 50% of the celiac population and can affect protein synthesis leading to growth arrest [44]; thus, a zinc supplementation between 25–40 mg/day is recommended [60]. In the present study, *Laminaria* incorporation in pasta led to an increase of Zn from 4.8% to 6.3% RDA. As GF products are usually lower in magnesium, some celiac patients need additional magnesium supplement of 200–300 mg/day in the form of magnesium oxide or magnesium chloride, whereas others can improve magnesium levels through dietary means [60]. *Laminaria* incorporation in GF pasta rendered an increase from 2.5% to 4.5% RDA of Mg, promoting an additional source of Mg to the diet.

*Laminaria* incorporation in pasta led also to the increase in manganese (13.8% to 16.7% RDA) and copper (10.4% to 19.4% RDA) contents. Manganese is an essential trace elemental needed for normal function and regeneration of the central nervous system and is linked to osteoporosis, epilepsy, and altered lipid and carbohydrate metabolism [62]. Regarding copper, *Laminaria* incorporation in GF pasta almost doubled its content compared to control pasta (Table 2), much higher than the values reported by Orecchio et al. [59] for commercial rice noodles and pasta. This mineral is needed for adequate growth, cardiovascular integrity, lung elasticity, neovascularization, neuroendocrine function, and iron metabolism [63].

*Laminaria ochroleuca* is an excellent source of iodine, and therefore an alternative to fulfilling the daily iodine needs of consumers (0.15 mg I/day), and especially in celiac sufferers, as they are more susceptible to thyroid disease than non-celiacs [56]. However, one should be aware that PL pasta would provide 155% of the daily recommended amount of iodine. Although iodine ingestion above the recommended threshold is generally well-tolerated, certain susceptible individuals, including those with pre-existing thyroid disease and the elderly, risk developing iodine-induced thyroid dysfunction [64].

### 3.4. Effect of Laminaria Processing on the Presence of Phytochemicals in the Pasta

The total phenolic content (TPC) and in vitro antioxidant activity (AA) of pastas was performed by DPPH and ABTS methods (Figure 4). Moreover, considering that these foods are consumed after thermal processing, and that cooking induces great changes in texture, molecule structure, content and availability [23], determinations were repeated also on cooked samples.

An earlier research study [28] showed that *Laminaria ochroleuca* and its autohydrolysis liquid extract had considerable high phlorotannin content. These compounds were marine polyphenols only produced by brown algae [65]. In this sense, the total phenolic content of all pasta samples were determined, including the control.

The TPC of raw pastas ranged from 1.87–3.28 mg GAE/g, with the control showing similar TPC than PP pasta, much higher than the values reported by Prabhasankar et al. [21] for wheat pasta with *Undaria pinnatifida* (0.56 ± 0.02 mg GAE/g). Besides the differences in extraction methods used in both studies, the main issue was the raw materials that composed the control pasta, which was produced with rice flour and *Psyllium* husk, both known for having phenolics [66,67]. The higher TPC present in PL pasta was due to the processing of the alga (autohydrolysis) prior to pasta making. Phenolic compounds are usually present in bound states as conjugates with sugars, fatty acids, or proteins. During autohydrolysis of *L. ochroleuca*, the hydro-soluble phenolic compounds were released into the liquor, becoming more available for extraction and quantification after incorporation in pasta. According to Rajauria et al. [68], the increase in phenolic compound content after hydrothermal treatment, due to the disassociation of the complexes, followed by some polymerisation of the phenolics, may be responsible for the increased antioxidant capacity. However, during cooking of pasta, part of the phenolics were leached into the cooking water, explaining the decrease in TPC and AA values. Prabhasankar et al. [69] also notes that phenolics, in the cooked control semolina pasta, were not significantly different from samples supplemented with *Sargassum marginatum.* However, in some cases, phenolics in cereal products are reported to increase with cooking, as the process can soften the hard structure and break cellular components, allowing an easier extraction from the matrix [23,48].

Regarding the antioxidant activity of raw pastas, both scavenging activity methods showed different trends. Each of the methods provided an estimate of the capacity that was dependent upon reaction time, method used, and the complexity of the reaction kinetics. On the other hand, interaction/polymerization of phenolic compounds may cause antioxidant capacity to be underestimated in fruit samples [70]. Scavenging activity of seaweed-incorporated pasta varied from 111-391 mmol TEAC/g, correspondent to 2.8%–5.2% RSA (radical scavenging activity), meaning that all samples showed weak antiradical effect. These results are similar to the ones reported for semolina pasta with *Undaria pinnatifida* [21], with fermented dough [71], and also for GF pasta with green mussel [72]. The higher AA (ABTS method) observed in PP pasta could be due to other phytochemicals rather than only phenolic compounds. Fucoxanthin was identified as the predominant pigment in brown seaweed, including *L. ochroleuca* [10]. Fucoxanthin, β-carotene, and violaxanthin are carotenoids found in seaweed that exhibit powerful antioxidant properties [73], which could explain the higher results of PP pasta. Another hypothesis to explain the increase in antioxidant features could be the presence of hydrolysed protein [74]. Unlike other carotenoids, which are commonly used as food colorants, fucoxanthin is not sold as a bulk food ingredient [75]. Its instability due to oxidation and high extraction costs could be an opportunity for the development of *Laminaria*-enriched products.

### 3.5. Mechanical Properties of Pasta

#### 3.5.1. Rheology Characterization of Pasta Samples

**Figure 5:**
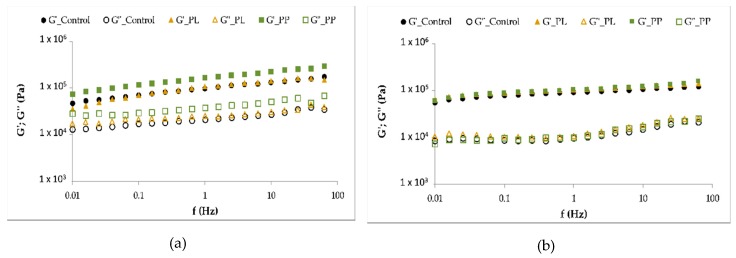
Mechanical spectra of raw (**a**) and cooked (**b**) gluten-free (GF) pastas: with *L. ochroleuca* liquid extract (PL), alga purée (PP), and control (without alga). Closed symbols—storage modulus, G’; open symbols—loss modulus, G”.

The results from the small amplitude dynamic rheology measurements (Figure 5) of the raw and cooked pastas were expressed in terms of storage (G’) and loss (G’’) moduli.

For both raw and cooked pastas, the storage modulus values (G’) were higher than those of the loss modulus (G’’), which revealed the elastic nature predominance in the studied samples. Similar rheology behaviour was identified by other authors on gluten-free formulations made with rice flour [76]. Raw pastas showed a rheology behaviour slightly dependent on the frequency, especially at higher frequency values, and no differences were found between control and pastas with *Laminaria* addition (PL, PP).

After cooking, all pastas became more structured and stable, evidenced by the less frequency dependence of the viscoelastic moduli (Figure 5b). They also presented similar rheology behaviour with a minimum in G’’, which indicated a second degree of structuring due to the entanglement of the biomolecules that comprise the food matrix [77]. Microstructure studies [69] revealed that the incorporation of seaweed *(Sargassum marginatum)* enhances the gluten network of pasta up to 2.5%, which resulted in improved pasta quality. Although PP pasta also had 2.5% (*w/w*) of seaweed incorporation, a different trend was observed. Unlike gluten-based foods, the cooked structure of GF foods (pasta included) is mainly due to starch physical modifications caused by the gelatinization process, forming a continuous phase of solubilized amylose and/or amylopectin [76]. Besides the positive role of starch on pasta structuring, we should also consider the role played by fibre, which is reported to disrupt the protein matrix in durum wheat pasta and noodles [78,79], and is consistent with rheology behaviour of pastas upon cooking (Figure 5).

#### 3.5.2. Texture Properties of Pasta Samples

The texture properties of the pasta samples were evaluated before and after cooking, by both penetration and tension tests (Table 3).

In raw pasta, *Laminaria* addition contributed to a significant (*p* < 0.05) increase in PP firmness value, probably related to the high fibre content of this pasta. After cooking, the firmness decreased to values in the range of 2.25–2.76 N, similar to GF pasta with green mussel (1.30–2.01 N [72]). Raw PP was the less cohesive pasta, which explained the significant higher cooking loss (3%) when compared to the other samples. PP presented the highest firmness value of the analysed raw pastas, and the results were consistent with rheology measurements.

Adhesiveness increase in *Laminaria*-supplemented pastas (PL and PP) could be related to the increase in fibre content as reported by Bouasla et al. [80] for GF pasta with lentils, as fibre absorbs water and can become adhesive. However, after cooking, they showed very low stickiness values, especially PL pasta, probably resulting from a different conformation on the hydrocolloids caused by heat processing.

*Laminaria* addition caused a significant (*p* < 0.05) increase of pasta extensibility, which could be related to the synergy between alga biopolymers and *Psyllium*, already found in *Laminaria–Psyllium* gels [28].

### 3.6. Limitations and Future Work

Although the present study was not focused on the impact of incorporation of *L. ochroleuca* on the sensory quality of pasta, an informal sensory trial was performed. The consumers tested the product and stated that the taste was good (even with no salt added), and that the characteristic alga odour was less intense after cooking.

Studies on the bioavailability of the interesting compounds, namely polyphenols, minerals, and fibre, should be undertaken to evaluate the real functionally of the developed products. Also, in order to ensure consumer safety, the gluten content of pasta samples must be assessed.

## 4. Conclusions

This study intended to develop a GF fresh pasta that fulfils some of the requirements needed by the celiac population, and at the same time, adds value to local an under-exploited raw materials (*Laminaria ochroleuca* and rice flour from broken grains).

*Laminaria ochroleuca* showed promising potential to be valued, either in full or using its liquid extract, for pasta supplementation in terms of fibre and mineral contents. The GF pasta developed could bear nutrition claims for “source of fibre” and “fat-free” and showed interesting mechanical properties.

## Figures and Tables

**Table 1:** Centesimal composition of raw and cooked pasta with *Laminaria* liquid extract (PL) and *Laminaria* purée (PP), and control (without alga).

			Raw Pasta			Cooked Pasta		
			Control	PL	PP	Control	PL	PP
Moisture		(g/100 g)	50.3 ± 0.2 ^d^	49.2 ± 0.4 ^d,e^	48.3 ± 0.5 ^e^	65.4 ± 1.4 ^b^	62.9 ± 0.9 ^c^	67.6 ± 1.1 ^a^
Ash		(g/100 g, d.b)	0.5 ± 0.0 ^e^	1.3 ± 0.1 ^b^	1.9 ± 0.1 ^a^	0.4 ± 0.0 ^e^	0.8 ± 0.0 ^d^	1.1 ± 0.1 ^c^
Lipids			2.2 ± 0.2 ^a^	0.7 ± 0.0 ^c^	1.7 ± 0.3 ^a,b^	0.6 ± 0.2 ^c^	0.9 ± 0.1 ^c^	1.1 ± 0.1 ^b,c^
Protein			7.7 ± 0.2 ^c^	8.2 ± 0.2 ^a^	8.3 ± 0.2 ^a^	7.7 ± 0.0 ^b,c^	8.2 ± 0.1 ^a,b^	8.1 ± 0.0 ^a,b,c^
Fibre	Insoluble		-	-	-	4.8 ± 0.4 ^b^	4.4 ± 0.4 ^b^	7.1 ± 0.1 ^a^
	Soluble		-	-	-	0.9 ± 0.3 ^b^	1.4 ± 0.5 ^b^	2.4 ± 0.2 ^a^
	Total		-	-	-	6.1 ± 0.7 ^b^	5.6 ± 0.8 ^b^	9.5 ± 0.3 ^a^
Minerals	Na	(mg/100 g, d.b)	74.1 ± 2.8 ^e^	145.7 ± 3.1 ^c^	229.1 ± 4.8 ^a^	70.0 ± 3.3 ^e^	110.4 ± 2.6 ^d^	159.4 ± 0.3 ^b^
	K		325.3 ± 3.3 ^e^	558.6 ± 3.7 ^b^	761.1 ± 3.4 ^a^	272.4 ± 4.4 ^f^	394.4 ± 7.7 ^d^	495.2 ± 8.8 ^c^
	Ca		7.4 ± 1.6 ^c^	14.6 ± 1.0 ^b^	50.3 ± 1.9 ^a^	9.9 ± 1.8 ^c^	15.0 ± 0.5 ^b^	47.3 ± 1.8 ^a^
	Mg		28.3 ± 0.2 ^e^	38.9 ± 0.4 ^c^	55.1 ± 1.1 ^a^	27.6 ± 0.5 ^e^	34.3 ± 0.1 ^d^	51.9 ± 0.6 ^b^
	P		115.8 ± 2.3 ^b^	123.2 ± 1.1 ^a^	121.9 ± 0.5 ^a^	109.5 ± 0.7 ^c^	110.0 ± 1.5 ^c^	116.9 ± 0.9 ^b^
	S		109.0 ± 4.2 ^c^	126.2 ± 1.6 ^b^	144.8 ± 1.4 ^a^	104.7 ± 1.8 ^c^	121.3 ± 2.8 ^b^	150.3 ± 3.0 ^a^
	Fe		0.7 ± 0.4 ^b^	0.5 ± 0.3 ^b^	0.5 ± 0.2 ^b^	1.2 ± 0.1 ^b^	3.9 ± 0.7 ^a^	1.5 ± 0.4 ^b^
	Cu		0.3 ± 0.3 ^b^	1.1 ± 0.4 ^a,b^	1.7 ± 0.5 ^a^	0.3 ± 0.1 ^b^	0.4 ± 0.2 ^b^	0.6 ± 0.0 ^b^
	Zn		1.1 ± 0.0 ^a^	1.6 ± 0.3 ^a^	1.4 ± 0.3 ^a^	1.3 ± 0.1 ^a^	1.7 ± 0.1 ^a^	1.6 ± 0.4 ^a^
	Mn		0.8 ± 0.1 ^a^	0.8 ± 0.1 ^a^	0.8 ± 0.1 ^a^	0.8 ± 0.1 ^a^	0.9 ± 0.2 ^a^	0.7 ± 0.1 ^a^
	I		0.1 ± 0.0 ^e^	0.8 ± 0.0 ^a^	0.5 ± 0.0 ^c^	0.1 ± 0.0 ^e^	0.6 ± 0.0 ^b^	0.4 ± 0.0 ^d^

Data are presented as mean ± SD. Different letters in the same parameter (e.g., ash raw and cooked) correspond to significant differences (*p* < 0.05, one-way ANOVA, *post-hoc* Tukey test). *Laminaria ochroleuca* liquid extract (PL), *Laminaria ochroleuca* purée (PP).

**Table 2:** Mineral content of cooked pasta with *Laminaria* liquid extract (PL) and *Laminaria* purée (PP), and control (without alga). The % values in the columns indicate the recommended daily allowance (RDA) correspondent to the mineral content.

Minerals (mg/100 g)	Control	% RDA_Control_	PL	% RDA_PL_	PP	% RDA_PP_
K	94.3	4.7	146.3	7.3	160.4	8.0
Ca	3.4	0.4	5.6	0.7	15.3	1.9
Mg	9.5	2.5	12.7	3.4	16.8	4.5
P	37.9	5.4	40.8	5.8	37.9	5.4
Fe	0.4	3.0	1.4	10.3	0.5	3.5
Cu	0.1	10.4	0.1	14.8	0.2	19.4
Zn	0.4	4.5	0.6	6.3	0.5	5.2
Mn	0.3	13.8	0.3	16.7	0.2	11.3
I	0.0	15.0	0.2	155.6	0.1	92.2

**Table 3:** Texture parameters of raw and cooked pasta.

			Control	PL	PP
**Raw Pasta**	Firmness (N)	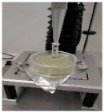	3.2 ± 0.5 ^b^	3.6 ± 0.6 ^b^	5.8 ± 0.4 ^a^
	Adhesiveness (N·s)		−0.2 ± 0.1 ^a^	−0.5 ± 0.1 ^b^	−0.5 ± 0.1 ^b^
	Cohesiveness		0.5 ± 0.0 ^a^	0.5 ± 0.0 ^a^	0.3 ± 0.0 ^b^
**Cooked Pasta**	Firmness (N)	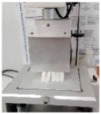	2.8 ± 0.3 ^a^	2.4 ± 0.3 ^b^	2.3 ± 0.3 ^b^
	Stickiness (N)	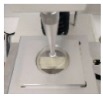	1.5 ± 0.4 ^a^	0.7 ± 0.2 ^b^	1.4 ± 0.4 ^a^
	Rmax (N)	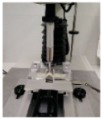	0.6 ± 0.1 ^a,b^	0.7 ± 0.1 ^a^	0.6 ± 0.1 ^b^
	ERmax (mm)		4.6 ± 1.4 ^a^	5.6 ± 1.3 ^b^	5.5 ± 1.1 ^b^

Data are presented as mean ± SD. Different superscript letters in the same parameter correspond to significant differences (*p* < 0.05, one-way ANOVA, *post-hoc* Tukey test).
